# Phylogeny and mitochondrial gene order variation in Lophotrochozoa in the light of new mitogenomic data from Nemertea

**DOI:** 10.1186/1471-2164-10-364

**Published:** 2009-08-06

**Authors:** Lars Podsiadlowski, Anke Braband, Torsten H Struck, Jörn von Döhren, Thomas Bartolomaeus

**Affiliations:** 1 Abteilung Evolutionsbiologie, Institut für Evolutionsbiologie und Ökologie, Universität Bonn, Germany; 2 Abteilung Vergleichende Zoologie, Institut für Biologie, Humboldt-Universität Berlin, Germany; 3 Arbeitsgruppe Zoologie, FB05 Biologie/Chemie, Universität Osnabrück, Germany

## Abstract

**Background:**

The new animal phylogeny established several taxa which were not identified by morphological analyses, most prominently the Ecdysozoa (arthropods, roundworms, priapulids and others) and Lophotrochozoa (molluscs, annelids, brachiopods and others). Lophotrochozoan interrelationships are under discussion, e.g. regarding the position of Nemertea (ribbon worms), which were discussed to be sister group to e.g. Mollusca, Brachiozoa or Platyhelminthes. Mitochondrial genomes contributed well with sequence data and gene order characters to the deep metazoan phylogeny debate.

**Results:**

In this study we present the first complete mitochondrial genome record for a member of the Nemertea, *Lineus viridis*. Except two *trnP *and *trnT*, all genes are located on the same strand. While gene order is most similar to that of the brachiopod *Terebratulina retusa*, sequence based analyses of mitochondrial genes place nemerteans close to molluscs, phoronids and entoprocts without clear preference for one of these taxa as sister group.

**Conclusion:**

Almost all recent analyses with large datasets show good support for a taxon comprising Annelida, Mollusca, Brachiopoda, Phoronida and Nemertea. But the relationships among these taxa vary between different studies. The analysis of gene order differences gives evidence for a multiple independent occurrence of a large inversion in the mitochondrial genome of Lophotrochozoa and a re-inversion of the same part in gastropods. We hypothesize that some regions of the genome have a higher chance for intramolecular recombination than others and gene order data have to be analysed carefully to detect convergent rearrangement events.

## Background

Starting about 25 years ago molecular phylogenetic approaches established a new system of animal taxonomy [1,2]. Bilateria are split into three major subtaxa, the traditional Deuterostomia and two recently established groups, which were founded initially by molecular evidence: the Ecdysozoa (combining arthropods with nemathelminth taxa like nematodes, priapulids etc.) and the Lophotrochozoa (comprising the taxa formerly combined in Spiralia, except Arthropoda, but additionaly including the lophophorate taxa Brachiopoda, Phoronida and Ectoprocta). Despite controversy about the specific position of some taxa, these three major groups now seem to be well established and are frequently recovered in analyses of different molecular datasets like ribosomal RNAs [3-6], mitochondrial genomes [7-9] and EST datasets [10-13].

The lophotrochozoan taxon Nemertea (ribbon worms) comprises about 1150 free-living species, most of which inhabit marine environments, but a few species also occur in freshwater and even in terrestrial habitats [14]. Morphological characters like the acoelomate organisation, the architecture of the nervous system, the sense organs and the protonephridial excretory structures were arguments for the traditional placement of Nemertea close to the Platyhelminthes (reviewed in [15]), while a trochophora-like larva with a prototroch gives some evidence for an inclusion into Trochozoa [16]. Special features like the closed circulatory system (in an acoelomat body cavity!) and the retractable proboscis, serving for prey catching, are apomorphies which clearly support monophyly of the Nemertea [17].

Nemerteans are among the first acoelomates to be brought together with coelomates, providing the ground for the 'new view' of animal phylogeny [18]. Meanwhile further molecular analyses came up with diverse hypotheses for their phylogenetic position. Depending on datasets and methods used for phylogenetic inference the propsed sister group of Nemertea was Platyzoa [19], Mollusca [13,20,21], Molluca + Annelida (= Neotrochozoa) [22,23]. Recent approaches with large datasets from EST libraries added another hypothesis: in the phylogenetic analyses of Dunn et al. [12] and Helmkampf et al. [24] Nemertea cluster with Brachiopoda and Phoronida.

Animal mitochondrial genomes provide a large set of orthologous sequence data which are often used in phylogenetic analyses from population to phylum level. In addition to sequence information several other features are used to support phylogenetic hypotheses, e.g. gene order rearrangements, derived secondary structure of rRNAs and tRNAs, changes in genetic code (for a review see [25]). Mitochondrial gene order data had an early impact on formation of the Lophotrochozoa hypothesis: Stechmann and Schlegel [26] demonstrated a highly similar gene order when comparing the brachiopod *Terebratulina retusa *and the mollusc *Katharina tunicata*, giving a strong argument in favour of the Lophotrochozoa hypothesis. The main difference between the two species is one big inversion covering about half of the entire genome. Gene order of the partial mitochondrial genome from the nemertean *Cephalothrix rufifrons *is not much different from that of *Katharina *and *Terebratulina *[20].

In this study we present the first complete mitochondrial genome record for a member of the Nemertea, *Lineus viridis*. We use mitochondrial gene order and sequence data to evaluate the phylogenetic position of Nemertea. Furthermore, we discuss mitochondrial gene order data among Lophotrochozoa and conclude that specific inversions may have occurred independently in different taxa, probably providing a rare example of homoplasious change of gene order.

## Results and Discussion

### General features of the mitochondrial genome of Lineus viridis

**Figure 1:**
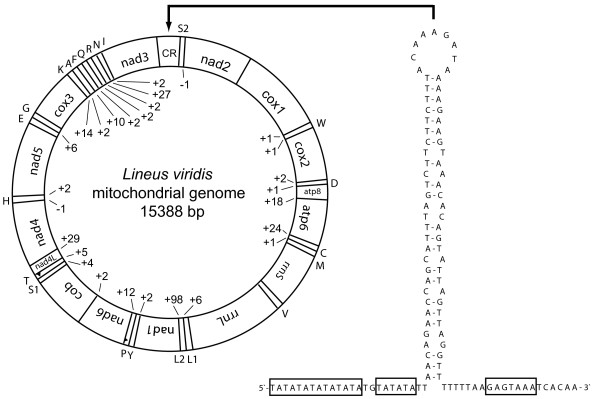
**Circular map of the mitochondrial genome of *Lineus viridis *and stem-loop structure of the control region**. tRNA genes are represented by their corresponding amino acid one letter abbreviation. Except trnT and trnP all genes are on the same strand and are oriented (5'-3') in clockwise manner. Numbers (+/-) depict noncoding nucleotides between genes or overlapping nucleotides, respectively. The stem-loop structure is annotated minus-strand like, to show signal sequences (boxed) similar to that found in arthropod control region. The depicted region correspondes to c14260 – c14150 of the GenBank record.

All 37 genes usually present in bilaterian animals are found in the mitochondrial genome of *L. viridis *(GenBank accession number FJ839919). All protein-coding and ribosomal RNA genes, as well as all but two tRNA genes (*trnP, trnT*) are found on the same strand, therefore defined as plus-strand (Table 1, Figure 1). This preference of one strand is also found in other lophotrochozoan taxa (Annelida, Brachiopoda, Acanthocephala, Platyhelminthes) [27], as well as in Tunicata [28]. The size of the genome (15388 bp) is well in the range of other lophotrochozoans [27]. The complete genome has an AT content of 65.8%, which is not significantly different from other Lophotrochozoa like *Lumbricus terrestris *(62%, [29]), *Katharina tunicata *(69%, [30]) or *Terebratulina retusa *(57%, [26]). Plus-strand shows a strong GC-skew (0.306) and AT-skew (-0.352), as the nucleotide composition is clearly biased towards G and T (A: 21.3%, C: 11.9%, G: 22.4%, T: 44.4%).

**Table 1:** Genome organisation of *Lineus viridis *(complete length: 15388 bp).

***Gene***	***Strand***	***Position*** ***(start – end)***	***Length*** ***(nuc.)***	***GC-skew***	***Start-codon***	***Stop-codon***	***Intergenic nucleotides***
*cox1*	+	1 – 1533	1533	0.236	ATG	TAG	1
*trnW*	+	1535 – 1599	65				1
*cox2*	+	1601 – 2287	687	0.311	ATG	TAA	2
*trnD*	+	2290 – 2353	64				1
*atp8*	+	2355 – 2513	159	0.367	ATG	TA	18
*atp6*	+	2532 – 3224	693	0.350	ATG	TAG	24
*trnC*	+	3249 – 3310	62				1
*trnM*	+	3312 – 3376	65				*
*rrnS *(12S)*	+	3377 – 4209	833	0.254			*
*trnV*	+	4210 – 4275	66				*
*rrnL *(16S)*	+	4276 – 5580	1305	0.322			*
*trnL*-CUN	+	5581 – 5649	69				6
*trnL*-UUR	+	5656 – 5721	66				98
*nad1*	+	5820 – 6750	931	0.295	ATG	T	2
*trnY*	+	6753 – 6818	66				0
*trnP*	-	6819 – 6883	65				12
*nad6*	+	6896 – 7354	459	0.446	ATG	TAG	2
*cob*	+	7357 – 8491	1135	0.230	ATG	T	0
*trnS*-UCN	+	8492 – 8559	68				4
*trnT*	-	8564 – 8628	65				5
*nad4L*	+	8634 – 8942	309	0.505	ATG	TAA	-7
*nad4*	+	8936 – 10287	1352	0.327	ATG	TAA	-1
*trnH*	+	10287 – 10351	65				2
*nad5*	+	10354 – 12086	1733	0.377	ATG	TA	0
*trnE*	+	12087 – 12149	63				6
*trnG*	+	12156 – 12219	64				0
*cox3*	+	12220 – 12999	780	0.272	ATG	TAA	14
*trnK*	+	13014 – 13082	69				2
*trnA*	+	13085 – 13150	66				11
*trnF*	+	13162 – 13225	64				3
*trnQ*	+	13229 – 13296	68				2
*trnR*	+	13299 – 13356	58				2
*trnN*	+	13359 – 13424	66				27
*trnI*	+	13452 – 13519	68				2
*nad3*	+	13522 – 13887	366	0.407	ATG	TAG	415
Major NCR*		13888 – 14302	415	0.210			*
*trnS*-AGN	+	14303 – 14372	70				-1
*nad2*	+	14372 – 15388	1017	0.365	ATG	TAA	0

* start and stop position of ribosomal RNA and NCR according to adjacent gene boundaries

A total of 676 non-coding nucleotides is found in the mt genome, comprising about 4.4% of the complete sequence. The major non-coding region is found between *nad3 *and *trnS*(SGN)/*nad2*, and has a slightly higher AT content (68.8%) than the remaining genome. Other lophotrochozoans with a similar gene order as *Lineus *(including the nemertean *Cephalothrix rufifrons*, [20]) do not have a non-coding sequence at that position. Near the 3'-end there is a 67 bp segment having the potential of forming a stem-loop structure. Figure 1 shows this structure and flanking sequences in minus-strand annotation, to show the flanking regions with putative signal sequences similar to that described from arthropod control regions [31,32]. The second-largest non-coding part is found between *trnL*(UUR) and *nad1 *(98 bp), which has a higher AT content than other parts of the genome (74.5%). Other non-coding regions >10 bp are found between *atp6 *and *trnC *(24 bp), *trnY *and *trnP *(12 bp), *trnG *and *cox3 *(14 bp), trnA and trnF (11 bp) and *trnR *and *trnN *(27 bp). Between *nad4 *and *trnH *there seems to be an overlap of 10 nucleotides.

### Protein-coding genes and rRNAs

All protein-coding genes use exclusively ATG as start codon, while stop codon TAA (5×) and TAG (4×) are used almost equally often (Table 1). Four genes have incomplete stop codons (TA-, T-), a feature often found in animal mitochondrial genomes. Incomplete stop codons are probably subject to post-transcriptional polyadenylation [33]. All protein-coding genes are encoded on the plus-strand and show a positive GC-skew, ranging from 0.236 in *cox1 *to 0.505 in *nad4L*. There is a trend for higher GC-skew in usually less conserved sequences like *nad3*, *nad4*, *nad5*, compared to more conserved genes like *cox1-3*, *cob*. The two ribosomal RNA genes (16S, 12S) are similar in length to those from other lophotrochozoan taxa, and as in many bilaterians, both are separated by *trnV*.

### Transfer RNAs

**Figure 2:**
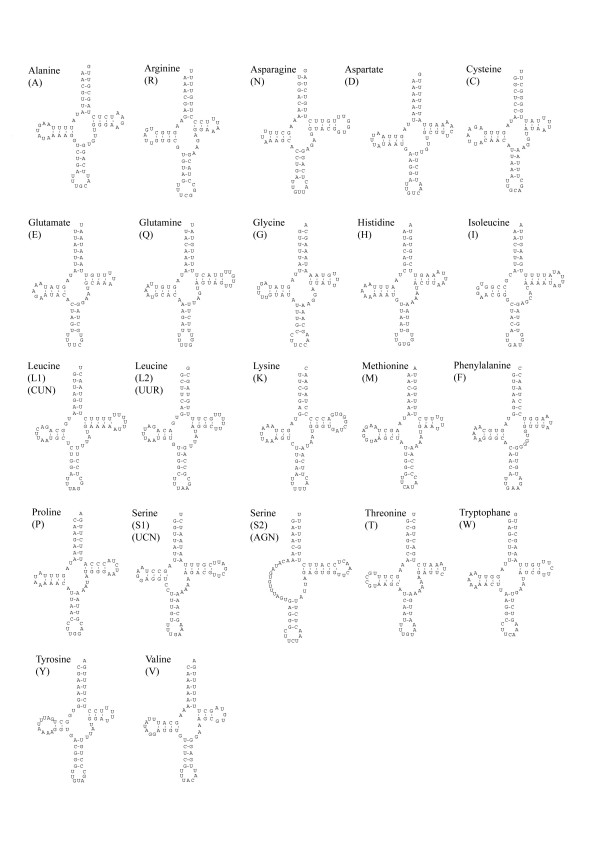
**Putative secondary structures of the 22 tRNAs identified in the mitochondrial genome of *Lineus viridis***.

The set of 22 tRNA genes typical for Bilateria were found in the mitochondrial genome of *Lineus viridis *(Figure 2). 21 of them can be folded into the typical cloverleaf secondary structure. The cloverleaf structure of tRNA-Ser(AGN) misses the DHU-arm, which is missing in most metazoan species, and is probably lost early in animal evolution [34]. A few mismatches are found in the acceptor stem of tRNA-His, tRNA-Lys, tRNA-Leu(UUR), and tRNA-Phe, as well as in the anticodon stem of tRNA-Glu and tRNA-Leu(CUN).

### Mitochondrial gene order in Lophotrochozoa

**Figure 3:**
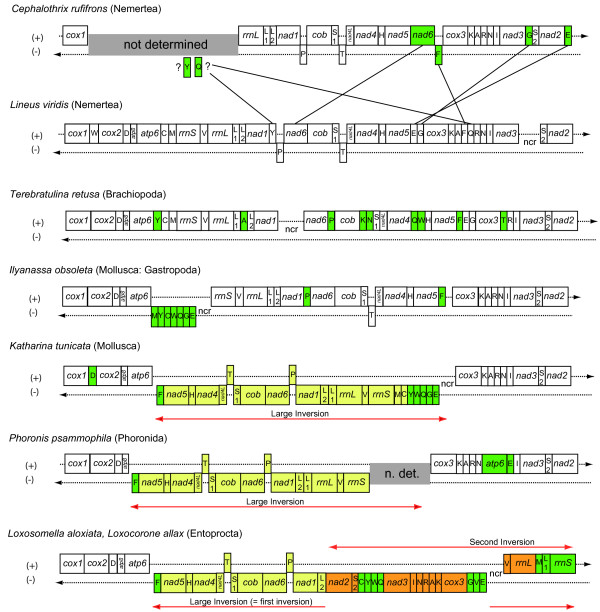
**Mitochondrial gene order of Nemertea and selected lophotrochozoan species**. Colour coded genes show different positions from that seen in Lineus viridis, according to transpositions (green) or inversions (yellow, orange). The yellow inversion is a potential synapomorphy. tRNA genes are abbreviated by their amino acids (one letter code). Upper genes are plus-strand encoded, lower genes are minus-strand encoded. Gene orders according to the following references: Cephalothrix [20], Terebratulina [26], Ilyanassa [38], Katharina [30], Phoronis [42], Entoprocta [43].

Gene order is not conserved in Nemertea, as the partial mt genome of *Cephalothrix rufifrons *[20] and the complete mt genome of *Lineus viridis *presented here differ in the position of *nad6 *and five tRNA genes (Figure 3). We assume that *Cephalothrix *shows the more derived condition among Nemertea, as the adjacency of *nad6 *and *cob *is very common in lophotrochozoan and also arthropod mitochondrial genomes. Therefore the condition *nad1-nad6-cob*, as observed in *Lineus *is likely the plesiomorphic state in Nemertea. As well the relative positions of most of the tRNA genes are conserved in *Lineus *and other non-nemertean taxa. The only exception is *trnF*, which is in a derived position in *Lineus *and in the ancestral position in *Cephalothrix*. *Lineus *is a member of the Heteronemertea, while *Cephalothrix *is a member of the Palaeonemertea, a group which is thought to be the sister group to the remaining Nemertea [35] and which has many ancestral characters compared to other Nemertea. It is another example of the fact that a taxon showing ancestral states for many characters may as well show derived states in other character complexes.

Gene order of *Lineus viridis *is very similar to that of some other lophotrochozoan taxa. Most of the differences between lophotrochozoan taxa concern translocations of tRNA genes, which seem to be more "mobile" than the larger genes [32,36]. Analysis of relative positions of tRNA genes yielded no phylogenetic informative character (data not shown), so we focused on the relative positions of the protein-coding and rRNA genes. Their gene order is identical in *Lineus*, the brachiopod *Terebratulina retusa *[26], and some gastropods, e.g. *Conus textile *[37], *Ilyanassa obsoleta *[38], *Thais clavigera *(GenBank NC_010090), and *Lophiotoma cerithiformis *[39]. Turbeville and Smith [20] also analysed mitochondrial gene order of a partial genome of the nemertean *Cephalothrix rufifrons*. Their gene adjacency analyses clustered *Cephalothrix *with molluscs, preferentially *Haliotis*, but the brachiopod *Terebratulina *was missing in their analyses. Other molluscs like the gastropod *Haliotis rubra *[40], the polyplacophoran *Katharina tunicata *[26] and the cephalopod *Octopus vulgaris *[41] show a similar gene order, but distinguished by a large inversion of about half the mt genome (Figure 3). The segment spanning from *trnF *to *trnE *(adjacent to the control region) is found in opposite direction than the remainder of the genome. Due to the broader distribution among Mollusca (Polyplacophora, Gastropoda, Cephalopoda) it is most parsimonious to assume the gene order of *Katharina *and *Octopus *(= with inversion) to be ancient within molluscs and to interpret gene order in the gastropods *Conus*, *Ilyanassa *and *Thais *to be secondarily re-inverted (other molluscs like Scaphopoda and Bivalvia show strongly derived gene orders compared to the mentioned species). Besides molluscs a similar inversion is seen in the mt genome of *Phoronis psammophila *[42] and, secondarily complicated by another inversion, in the Entoprocts *Loxosomella *and *Loxocorone *[43]. This inversion may be a synapomorphy of Phoronida + Entoprocta + Mollusca. However, there is no good support from sequence based analyses for a clade combining exclusively these three taxa (see below). Furthermore, an inversion similar to that described for Lophotrochozoa is found in Ecdysozoa, comparing arthropod and priapulid gene order [8]. Thus there is also reason to suspect some parts of the genome to be more often involved in rearrangements than others. In particular the mitochondrial control region may represent a region with "predetermined breaking points" in the mitochondrial genome, as there is non-coding sequence and no functional gene will be disrupted by a breakpoint. Besides its position the second breaking point cannot be further characterized by now. As there is a re-inversion in some gastropods, we cannot exclude that this inversion took place independently two or three times in Phoronida, Mollusca and Entoprocta. Nonetheless, it is reasonable to assume that the basal condition in Bilateria or at least Lophotrochozoa is to have all genes on the same strand – this is actually seen in Brachiopoda, Annelida, Platyhelminthes and Acanthocephalans.

### Phylogenetic analysis (of mitochondrial amino acid sequences)

**Figure 4:**
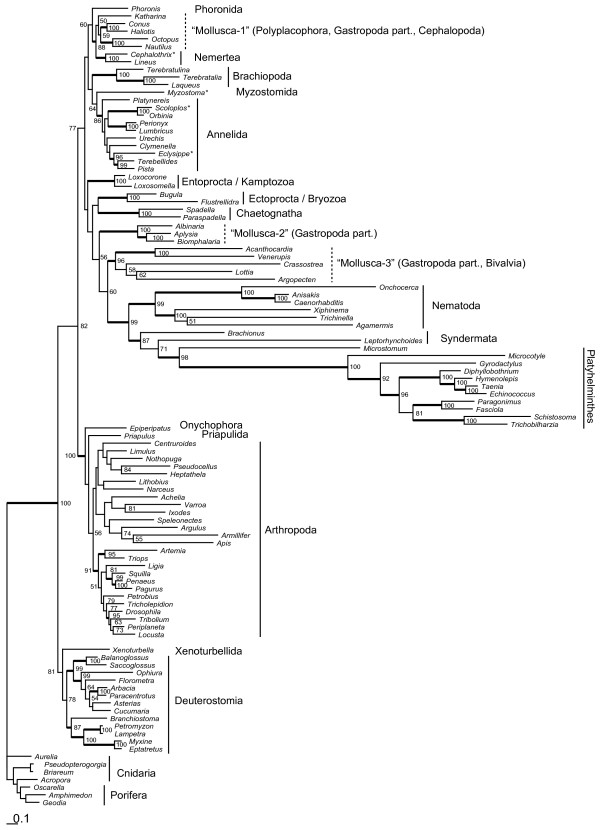
**Best tree from maximum likelihood analysis (RAxML, mtRev+G+I) with the 104 taxa dataset (concatenated amino acid alignments)**. Numbers indicate bootstrap percentages (>50%). Thick lines for clades indicate bootstrap support of at least 85%. Dotted lines depict taxa appearing as polyphyletic in our analysis. Scale bar depicts substitutions per site. For complete species names and accession numbers of GenBank entries see Additional file 1. Asterisks indicate taxa with incomplete mt genome records.

For phylogenetic analyses concatenated amino acid alignments from twelve mitochondrial protein-coding genes (all but the short and less conserved *atp8*) were built and analyzed by maximum likelihood and Bayesian methods. For a preliminary analysis a taxon set of 104 metazoan species was chosen. Seven species from Porifera and Cnidaria served as outgroup for rooting the Bilaterian tree. This large taxon set was analysed with a maximum likelihood approach (RAxML) and the best topology was tested by bootstrapping (Figure 4). Bilateria is split into three large clades: (1) Deuterostomia + Xenoturbella, (2) Arthropoda + Onychophora + Priapulida, (3) Lophotrochozoa with some long-branching taxa from other groups, prominently Nematoda. While many other molecular datasets favour Ecdysozoa hypothesis, thus a position of Nematoda with Arthropoda and Priapulida, our result seems to be artificial due to long-branch attraction. In our analysis Nematoda, Platyhelminthes, Syndermata and some subtaxa of Mollusca have the longest branches of all taxa and cluster together. Molluscan polyphyly is another strange effect of this problem. In the large dataset Nemertea are found to be sister group to short-branched taxa of Mollusca (a polyplacophoran, two gastropods and two cephalopods), with a bootstrap support of 88%. Other gastropod species and Bivalvia are found near the long-branching taxa of Nematoda, Syndermata and Platyhelmithes. Basal splits among Lophotrochozoa do not exceed moderate support in bootstrap analysis.

**Figure 5:**
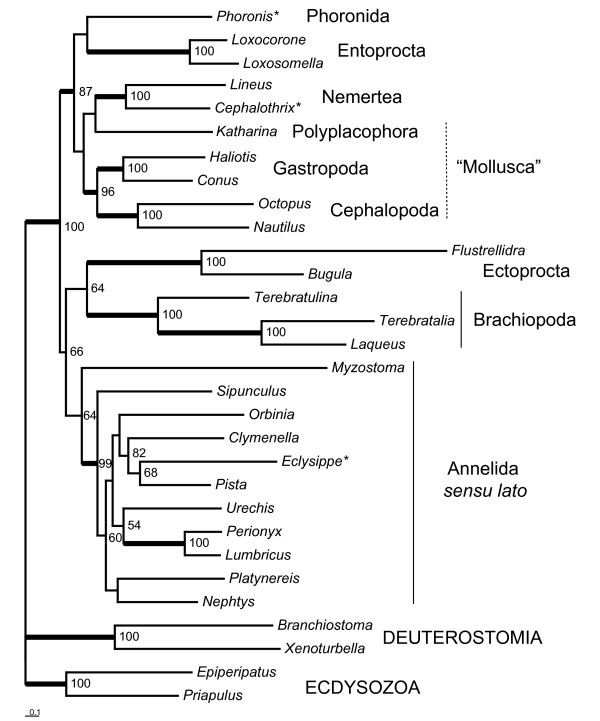
**Best tree from maximum likelihood analysis (RAxML, mtRev+G+I) with the 30 taxa dataset (concatenated amino acid alignments)**. Numbers indicate bootstrap percentage (RAxML, mtRev+G+I). Thick lines for clades indicate bootstrap support of at least 85%. Dotted lines depict taxa appearing as polyphyletic in our analysis. Scale bar depicts substitutions per site. For complete species names and accession numbers of GenBank entries see [Additional file 1]. Asterisks indicate taxa with incomplete mt genome records.

**Figure 6:**
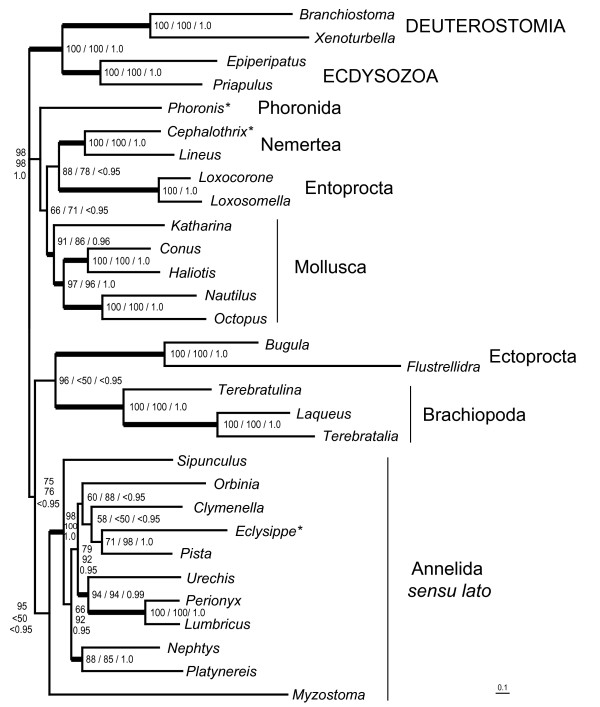
**Best tree from maximum likelihood analysis (Treefinder, mtZoa+G+I) with the 30 taxa dataset (concatenated amino acid alignments)**. Numbers next to nodes reflect edge support percentage (= LR-ELW) from Treefinder with mtZoa+G+I model (left or upper number), edge support percentage from Treefinder with mtRev+G+I model (middle number) and Bayesian posterior probability (BPP, mtRev+G+I, right or lower number). In the best tree of Treefinder with mtRev+G+I model Myzostoma clustered with Ectoprocta (edge support: 51%). The best tree from Bayesian analysis favoured another topology: Nemertea are sister group to Phoronida+Entoprocta (BPP: 1.0) and Myzostoma clustered with Ectoprocta (BPP: 1.0). Thick lines for clades indicate a combination of edge support above 85% and BPP above 0.95. Scale bar depicts substitutions per site. For complete species names and GenBank accession numbers see Additional file 1. Asterisks indicate taxa with incomplete mt genome records.

For more sophisticated analyses we used a smaller dataset of 26 lophotrochozoan species and four outgroup members from Deuterostomia and Ecdysozoa. We omitted Platyhelminthes, Nematoda, Syndermata and some of the molluscan taxa with long branches. As well we did not use sequences from Chaetognatha, due to their uncertain relation to Lophotrochozoa and we ignored sequences from the molluscs *Albinaria*, *Aplysia*, *Biomphalaria*, which did not cluster with the other molluscs in the first analysis. The best tree obtained by RAxML with mtRev+G+I (Figure 5) found the two nemertean species as sister group to the polyplacophoran mollusc *Katharina tunicata*, but without bootstrap support exceeding 50%. Thus, Mollusca again are not monophyletic under these parameters. The best tree from Treefinder analysis (Figure 6) with a model specified for lophotrochozoan taxa (mtZoa+G+I [44]) has a different topology, with Entoprocta being sister group to Nemertea, with moderate support from resampling analysis (edge support by LR-ELW: 88%). The five molluscan species form a monophylum with 91% edge support and are sister to the nemertean/entoprocta clade (LR-ELW: 66%). *Phoronis *is sister to that assemblage. The rest of the tree is similar to the RAxML tree. The best tree from Treefinder analysis with the mtRev+G+I (topology not shown, LR-ELW in Figure 6) model differs from that with mtZoa+G+I only in the position of *Myzostoma *as sister group to Ectoprocta. Here, support from LR-ELW for the Nemertea+Entoprocta relationship is 78%. The best tree of a Bayesian analysis (mtRev+G+I, topology not shown, BPP in Figure 6) of the same dataset resulted in a taxon combining Entoprocta and *Phoronis *as sister group to Nemertea (BPP: 1.0). Here, Mollusca is monophyletic (BPP: 0.96), while *Myzostoma *clustered with Ectoprocta (BPP: 1.0) instead of annelids as in the shown trees. The remaining tree topology is the same as in the Treefinder-mtZoa analysis. All four analyses favour a clade combining Phoronida, Entoprocta, Nemertea and Mollusca (RAxML/mtRev: 87%, Treefinder/mtZoa: 98%, Treefinder/mtRev: 98%, MrBayes/mtRev: 1.0). AU test of the RAxML analyses with constrained trees (Table 2) rejects the hypotheses of sister group relationships Nemertea+Annelida or Nemertea+Brachiopoda. Mollusca, Phoronida and Entoprocta cannot be excluded as possible sister groups to Nemertea according to that test.

**Table 2:** Hypothesis testing using the 30 taxa datset and constrained user trees.

**Tree**	**Log ML**	**AU test**
Best tree (Nemertea, Mollusca)	**-155485.329785**	**0.627**
(Nemertea, Entoprocta)	**-155486.866702**	**0.538**
(Nemertea, Phoronis)	**-155487.374372**	**0.439**
(Mollusca, Phoronis)	**-155505.665687**	**0.065**
(Phoronis, Brachiopoda, Entoprocta)	-155519.033604	0.030
(Phoronis, Brachiopoda)	-155530.837097	0.019
(Annelida, Mollusca)	-155558.159318	0.008
(Nemertea, Brachiopoda)	-155563.672164	0.001
(Nemertea, Annelida)	-155577.707414	1e-004

Dunn et al. [12], analysing a large EST dataset, found Entoprocta as sister group to the remaining taxa Mollusca, Annelida, Phoronida, Brachiopoda and Nemertea. Nemerteans are found to be sister group to Brachiopoda in one of their analyses, and sister group to a clade combining Brachiopoda and Phoronida in the second analysis (with a slightly reduced taxon set). The latter assemblage found support in some of their parameter settings. Here, Annelida sensu lato were the sister group of Nemertea, Brachiopoda and Phoronida, but only with moderate support. Struck & Fisse [13] found good support for Mollusca+Nemertea in Bayesian analyses of an amino acid alignment derived from EST data, while ML analyses were rather indifferent between Annelida and Mollusca as sister group to Nemertea. But these analyses did not include phoronid and brachiopod species. A partial mitochondrial genome of another nemertean species, *Cephalothrix rufifrons*, was previously published [20]. The corresponding phylogenetic analyses favoured an affinity to molluscs, which appeared paraphyletic in that study.

## Conclusion

Phylogenetic analyses of available mitochondrial sequence data (concatenated amino acid sequences) do not clearly resolve lophotrochozoan interrelationships, but favour a clade combining Nemertea, Mollusca, Phoronida and Entoprocta on one hand, Brachiopoda, Ectoprocta, Annelida, Sipuncula and Myzostomida on the other. Recent large analyses of EST datasets with similar taxon sampling came to other results. Mitochondrial gene order is very similar in Nemertea, some brachiopods and some molluscs, suggesting a shared ground pattern at least for a lophotrochozoan subtaxon. Phoronid and entoproct gene order is easily derivable from this ground pattern, while gene order of annelids and ectoprocts seems to be strongly derived, also in comparison to gene order of outgroup taxa from Ecdysozoa and Deuterostomia. In conclusion, none of the recent molecular based studies (mitochondrial genomes, EST approaches) found support for a relationship between Nemertea and Platyhelmithes, but the sister group to Nemertea remains an open question with more evidence for the candidates Mollusca, Phoronida, Entoprocta, Brachiopoda and less evidence for Annelida.

## Methods

### Animal samples and DNA extraction

Specimen of *Lineus viridis *were sampled on the island Sylt and fixed in 99.8% ethanol. DNA extraction was done with DNeasy Blood and Tissue kits (Qiagen, Hilden, Germany) according to manufacturers protocol for animal tissue.

### PCR and sequencing

Several standard PCR primer sets were tested to yield fragments of mitochondrial genes. Amplification was successful with the following primers: cox1: LCO-1490, 5'-GGT CAA CAA ATC ATA AAG ATA TTG G-3'; HCO-2198, 5'-TAA ACT TCA GGG TGA CCA AAA AAT CA-3' [45]; 16S: 16SarL, 5'-CGC CTG TTT AAC AAA AAC AT-3'; 16SbrH, 5'-CCG GTC TGA ACT CAG ATC ACG T-3' [46]. All PCRs were performed on Eppendorf Mastercycler and Mastercycler gradient. In these short-range PCR experiments Eppendorf 5-prime-Taq kit (Eppendorf, Germany) was used in 50 μl volumes (5 μl buffer; 1 μl dNTP mix, 10 μM; 0.25 μl Taq polymerase; 1 μl DNA, 40.75 μl water, 1 μl primer mix, 10 μM each). PCR products were purified using the Nucleospin kit (Macherey & Nagel). PCR conditions were: initial denaturation (94°C, 1 min), 40 cycles of denaturation (94°C, 30 sec), annealing (50°C, 30 sec), and elongation (68°C, 1 min), followed by a final elongation step (68°C, 1 min). These PCR products were sequenced using the Beckman-Coulter CEQ 8000 machine and DTCS kit (Beckman-Coulter) following the manufacturers protocol, except for using 10 μl volumes instead of 20 μl for the sequencing reaction.

These initial sequences along with mitochondrial sequences from an EST library generated by one of the authors [13,46] were used to design long-range PCR primers covering the complete mitochondrial genome of *Lineus viridis*. PCR was successfully performed with the primer sets Lv-cox1r (5'-CCA GTA CCA ACC AAA CCA GAC C-3')/Lv-16Sf (5'-AAA AGA TTG CGA CCT CGA TGT T-3) and Lv-16Sf (5'-AAA AGA TTG CGA CCT CGA TGT-3')/Lv-cox1r (5'-CCA GTA CCA ACC AAA CCA GAC C-3'). Long-range PCR was done with Takara LA Taq kit (Takara, distributed in Germany by MoBiTec) in 50 μl Volumes (34.5 μl water; 5 μl PCR buffer; 8 μl dNTP mix; 0.5 μl LA Taq; 1 μl primer-mix, 20 μM; 1 μl DNA). PCR conditions were: initial denaturation (94°C, 1 min), 40 cycles of denaturation (94°C, 30 sec), annealing (55°C, 1 min), and elongation (65°C, 12 min), followed by a final elongation step (65°C, 10 min). Long-range PCR products were purified using the Nucleospin kit (Macherey & Nagel) and subsequently used for a shotgun cloning approach (done in commission by Max Planck Institute of Molecular Genetics, Berlin).

### Sequence assemblage and annotation

Sequences were assembled using Bioedit [47]. Detection and annotation of tRNA genes was done making use of ARWEN [48] and tRNA scan SE [49]. Protein-coding and rRNA genes were firstly identified by BLAST search, then gene boundaries were detected in comparison with alignments of several lophotrochozoan taxa. Nucleotide composition was computed using Bioedit and GC- and AT-skew was determined by using the formulation of Perna and Kocher [50].

### Phylogenetic analysis

For phylogenetic analysis a concatenated dataset of mitochondrial amino acid alignments from 12 genes was built. The gene *atp8 *was excluded from the analysis, due to the fact that it is missing from many genomes (nematodes, platyhelminthes, chaetognaths), and that it is the smallest and least conserved of the protein-coding genes. Sequence data from 104 species, most of them with complete mt genome entries were retrieved from GenBank, for accession numbers see Additional file 1. Alignments were done with ClustalW [51] as implemented in Bioedit [47]. For the large dataset non-conserved sites were excluded from likelihood analyses making use of the Gblocks software [52], with the following parameter settings: minimum number of sequences for a conserved position: 55; minimum number of sequences for a flanking position: 55; maximum number of contiguous nonconserved positions: 8; minimum length of a block: 10; allowed gap positions: with half. In this case 2294 amino acid sites (= 49%) were recovered from the original dataset of 4654 amino acids. For maximum likelihood analysis, we used RAxML 7.0.4 [53,54] as offered on the CIPRES web portal. We choose mtRev+G+I, because mtRev was the only model derived from mitochondrial data available on this platform. We performed a search for the best tree and 100 bootstrap replicates. For more sophisticated analyses we chose a smaller dataset focussed on Lophotrochozoa (26 species) and using four species of Ecdysozoa and Deuterostomia representing the outgroup to Lophotrochozoa. Due to the better conservation among the alignments we used the complete alignments of twelve protein-coding genes and built a concatenated alignment with a final length of 3820 amino acids.

We used this smaller dataset to test different models in maximum likelihood analysis (mtRev, mtZoa), run a Bayesian analysis and performed hypothesis testing of alternative topologies. With the smaller dataset a partitioned model optimization was done in that we partitioned the dataset according to the 12 genes. Besides RAxML with mtRev+G+I (100 bootstrap runs) we used Treefinder v. Oct 2008 [55] to perform a maximum likelihood analysis with mtRev+G+I and the self implemented mtZoa+G+I model (each with LR-ELW, 1000 replications). The mtZoa model is optimzed for amino acid alignments from lophotrochozoan taxa [44]. In all likelihood analyses, models were the same for each partition but optimized in an unlinked manner between the partitions. In addition a Bayesian analysis was performed with MrBayes 3.1.2 [56]. 1,000,000 generations of two times four parallel chains were run, by sampling one tree out of thousand. According to the log likelihood plots 200 trees were discarded as burnin. Model settings were mtRev+G+I (unpartitioned due to time limitations). Hypothesis testing was done by computing best trees and per site likelihoods with RAxML (mtRev+G+I) for a set of constrained trees. Per site likelihoods were used to perform the AU-test [57], by making use of CONSEL 0.1j [58].

## Abbreviations

A: adenine; *atp6 *and *8*: genes encoding ATPase subunit 6 and 8; AU test: approximately unbiased test; BI: Bayesian inference; bp: base pairs; BPP: Bayesian posterior probability; *cox1-3*: genes encoding cytochrome oxidase subunits I-III; *cob*: gene encoding cytochrome b; C: cytosine; G: guanine; LR-ELW: edge support, local rearrangements (LR) around an edge of the best tree topology are analyzed for expected likelihood weights (ELW), yielding an approximation of the bootstrap value; ML: maximum likelihood; mt genome: mitochondrial genome; *nad1-6 *and *nad4L*: genes encoding NADH dehydrogenase subunits 1-6 and 4L; PCR: polymerase chain reaction; rRNA: ribosomal RNA; *rrnL*: large (16S) rRNA subunit (gene); *rrnS*: small (12S) rRNA subunit (gene); T: thymine; tRNA-Xyz (where Xyz is replaced by three letter amino acid code of the corresponding amino acid): transfer RNA; *trnX *(where *X *is replaced by one letter amino acid code of the corresponding amino acid), tRNA gene;

## Authors' contributions

LP conducted most of the PCR and sequencing experiments, annotation and phylogenetic analysis and wrote the main body of the manuscript. THS provided EST-sequences, and took part in discussion and manuscript writing. JD sampled and reared animals and performed DNA extraction and initial PCR experiments. AB provided substantial support in sequencing and phylogenetic analysis. TB was involved in discussion and manuscript writing. All authors read and approved the final manuscript.

## Supplementary Material

Additional file 1 Accession numbers. Click here for file

## References

[B1] Aguinaldo AM, Turbeville JM, Linford LS, Rivera MC, Garey JR, Raff RA (1997). Evidence for a clade of nematodes, arthropods and other moulting animals. Nature.

[B2] Halanych KM (2004). The new view of animal phylogeny. Annu Rev Ecol Syst.

[B3] Mallatt J, Giribet G (2006). Further use of nearly complete 28S and 18S rRNA genes to classify Ecdysozoa: 37 more arthropods and a kinorhynch. Mol Phylogenet Evol.

[B4] Mallatt JM, Garey JR, Shultz JW (2004). Ecdysozoan phylogeny and Bayesian inference: first use of nearly complete 28S and 18S rRNA gene sequences to classify the arthropods and their kin. Mol Phylogenet Evol.

[B5] Mallatt J, Winchell CJ (2002). Testing the new animal phylogeny: First use of combined large-subunit and small-subunit rRNA gene sequences to classify the protostomes. Mol Biol Evol.

[B6] Winchell CJ, Sullivan J, Cameron CB, Swalla BJ, Mallatt J (2002). Evaluating hypotheses of deuterostome phylogeny and chordate evolution with new LSU and SSU ribosomal DNA data. Mol Biol Evol.

[B7] Podsiadlowski L, Braband A, Mayer G (2008). The complete mitochondrial genome of the onychophoran Epiperipatus biolleyi reveals a unique transfer RNA set and provides further support for the ecdysozoa hypothesis. Mol Biol Evol.

[B8] Webster BL, Copley RR, Jenner RA, kenzie-Dodds JA, Bourlat SJ, Rota-Stabelli O (2006). Mitogenomics and phylogenomics reveal priapulid worms as extant models of the ancestral Ecdysozoan. Evol Dev.

[B9] Bourlat SJ, Juliusdottir T, Lowe CJ, Freeman R, Aronowicz J, Kirschner M (2006). Deuterostome phylogeny reveals monophyletic chordates and the new phylum Xenoturbellida. Nature.

[B10] Hausdorf B, Helmkampf M, Meyer A, Witek A, Herlyn H, Bruchhaus I (2007). Spiralian phylogenomics supports the resurrection of Bryozoa comprising Ectoprocta and Entoprocta. Mol Biol Evol.

[B11] Roeding F, Hagner-Holler S, Ruhberg H, Ebersberger I, von HA, Kube M (2007). EST sequencing of Onychophora and phylogenomic analysis of Metazoa. Mol Phylogenet Evol.

[B12] Dunn CW, Hejnol A, Matus DQ, Pang K, Browne WE, Smith SA (2008). Broad phylogenomic sampling improves resolution of the animal tree of life. Nature.

[B13] Struck TH, Fisse F (2008). Phylogenetic position of nemertea derived from phylogenomic data. Mol Biol Evol.

[B14] Gibson T (1995). Nemertean genera and species of the world: an annotated checklist of original names and description citations, synonyms, current taxonomic status, habitats and recorded zoogeographic distribution. J Nat Hist.

[B15] Nielsen C (2001). Animal Evolution Interrelationships of the living phyla.

[B16] Maslakova SA, Martindale MQ, Norenburg JL (2004). Vestigial prototroch in a basal nemertean, Carinoma tremaphoros (Nemertea; Palaeonemertea). Evol Dev.

[B17] Turbeville JM (2002). Progress in nemertean biology: development and phylogeny. Integr Comp Biol.

[B18] Turbeville JM, Field KG, Raff RA (1992). Phylogenetic position of phylum Nemertini, inferred from 18S rRNA sequences: molecular data as a test of morphological character homology. Mol Biol Evol.

[B19] Passamaneck Y, Halanych KM (2006). Lophotrochozoan phylogeny assessed with LSU and SSU data: Evidence of lophophorate polyphyly. Mol Phylogenet Evol.

[B20] Turbeville JM, Smith DM (2007). The partial mitochondrial genome of the Cephalothrix rufifrons (Nemertea, Palaeonemertea): Characterization and implications for the phylogenetic position of Nemertea. Mol Phylogenet Evol.

[B21] Helmkampf M, Bruchhaus I, Hausdorf B (2008). Multigene analysis of lophophorate and chaetognath phylogenetic relationships. Mol Phylogenet Evol.

[B22] Giribet G, Distel DL, Polz M, Sterrer W, Wheeler WC (2000). Triploblastic relationships with emphasis on the acoelomates and the position of Gnathostomulida, Cycliophora, Plathelminthes, and Chaetognatha: a combined approach of 18S rDNA sequences and morphology. Syst Biol.

[B23] Peterson KJ, Eernisse DJ (2001). Animal phylogeny and the ancestry of bilaterians: inferences from morphology and 18S rDNA gene sequences. Evol Dev.

[B24] Helmkampf M, Bruchhaus I, Hausdorf B (2008). Phylogenomic analyses of lophophorates (brachiopods, phoronids and bryozoans) confirm the Lophotrochozoa concept. Proc R Soc Lond B Biol Sci.

[B25] Boore JL (2006). The use of genome-level characters for phylogenetic reconstruction. Trends Ecol Evol.

[B26] Stechmann A, Schlegel M (1999). Analysis of the complete mitochondrial DNA sequence of the brachiopod Terebratulina retusa places Brachiopoda within the protostomes. Proc R Soc Lond B Biol Sci.

[B27] Valles Y, Boore JL (2006). Lophotrochozoan mitochondrial genomes. Integr Comp Biol.

[B28] Iannelli F, Griggio F, Pesole G, Gissi C (2007). The mitochondrial genome of Phallusia mammillata and Phallusia fumigata (Tunicata, Ascidiacea): high genome plasticity at intra-genus level. BMC Evol Biol.

[B29] Boore JL, Brown WM (1995). Complete sequence of the mitochondrial DNA of the annelid worm Lumbricus terrestris. Genetics.

[B30] Boore JL, Brown WM (1994). Complete DNA sequence of the mitochondrial genome of the black chiton, Katharina tunicata. Genetics.

[B31] Zhang DX, Szymura JM, Hewitt GM (1995). Evolution and structural conservation of the control region of insect mitochondrial DNA. J Mol Evol.

[B32] Kilpert F, Podsiadlowski L (2006). The complete mitochondrial genome of the common sea slater, Ligia oceanica (Crustacea, Isopoda) bears a novel gene order and unusual control region features. BMC Genomics.

[B33] Ojala D, Montoya J, Attardi G (1981). tRNA punctuation model of RNA processing in human mitochondria. Nature.

[B34] Haen KM, Lang BF, Pomponi SA, Lavrov DV (2007). Glass sponges and bilaterian animals share derived mitochondrial genomic features: a common ancestry or parallel evolution?. Mol Biol Evol.

[B35] Thollesson M, Norenburg JL (2003). Ribbon worm relationships: a phylogeny of the phylum Nemertea. Proc R Soc Lond B Biol Sci.

[B36] Bleidorn C, Eeckhaut I, Podsiadlowski L, Schult N, McHugh D, Halanych KM (2007). Mitochondrial genome and nuclear sequence data support myzostomida as part of the annelid radiation. Mol Biol Evol.

[B37] Bandyopadhyay PK, Stevenson BJ, Ownby JP, Cady MT, Watkins M, Olivera BM (2008). The mitochondrial genome of Conus textile, coxI-coxII intergenic sequences and Conoidean evolution. Mol Phylogenet Evol.

[B38] Simison WB, Lindberg DR, Boore JL (2006). Rolling circle amplification of metazoan mitochondrial genomes. Mol Phylogenet Evol.

[B39] Bandyopadhyay PK, Stevenson BJ, Cady MT, Olivera BM, Wolstenholme DR (2006). Complete mitochondrial DNA sequence of a Conoidean gastropod, Lophiotoma (Xenuroturris) cerithiformis: Gene order and gastropod phylogeny. Toxicon.

[B40] Maynard BT, Kerr LJ, McKiernan JM, Jansen ES, Hanna PJ (2005). Mitochondrial DNA sequence and gene organization in Australian backup abalone Haliotis rubra (leach). Mar Biotechnol.

[B41] Yokobori S, Fukuda N, Nakamura M, Aoyama T, Oshima T (2004). Long-term conservation of six duplicated structural genes in cephalopod mitochondrial genomes. Mol Biol Evol.

[B42] Helfenbein KG, Boore JL (2004). The mitochondrial genome of Phoronis architecta – comparisons demonstrate that phoronids are lophotrochozoan protostomes. Mol Biol Evol.

[B43] Yokobori S, Iseto T, Asakawa S, Sasaki T, Shimizu N, Yamagishi A (2008). Complete nucleotide sequences of mitochondrial genomes of two solitary entoprocts, Loxocorone allax and Loxosomella aloxiata: Implications for lophotrochozoan phylogeny. Mol Phylogenet Evol.

[B44] Rota-Stabelli O, Yang Z, Telford MJ (2009). MtZoa: a general mitochondrial amino acid substitutions model for animal evolutionary studies. Mol Phylogenet Evol.

[B45] Folmer O, Black M, Hoeh W, Lutz R, Vrijenhoek R (1994). DNA primers for amplification of mitochondrial cytochrome c oxidase subunit I from diverse metazoan invertebrates. Mol Mar Biol Biotechnol.

[B46] Palumbi SR (1996). What can molecular genetics contribute to marine biogeography? An urchin's tale. J Exp Mar Biol Ecol.

[B47] Hall TA (1999). BioEdit: a user-friendly biological sequence alignment editor and analysis program for Windows 95/98/NT. Nucl Acids Symp Ser.

[B48] Laslett D, Canback B (2008). ARWEN: a program to detect tRNA genes in metazoan mitochondrial nucleotide sequences. Bioinformatics.

[B49] Lowe TM, Eddy SR (1997). tRNAscan-SE: a program for improved detection of transfer RNA genes in genomic sequence. Nucleic Acids Res.

[B50] Perna NT, Kocher TD (1995). Patterns of nucleotide composition at fourfold degenerate sites of animal mitochondrial genomes. J Mol Evol.

[B51] Thompson JD, Higgins DG, Gibson TJ (1994). Clustal-W – Improving the Sensitivity of Progressive Multiple Sequence Alignment Through Sequence Weighting, Position-Specific Gap Penalties and Weight Matrix Choice. Nucleic Acids Res.

[B52] Castresana J (2000). Selection of conserved blocks from multiple alignments for their use in phylogenetic analysis. Mol Biol Evol.

[B53] Stamatakis A (2006). RAxML-VI-HPC: Maximum likelihood-based phylogenetic analyses with thousands of taxa and mixed models. Bioinformatics.

[B54] Stamatakis A, Hoover P, Rougemont J (2008). A rapid bootstrap algorithm for the RAxML web servers. Syst Biol.

[B55] Jobb G, von Haeseler A, Strimmer K (2004). TREEFINDER: a powerful graphical analysis environment for molecular phylogenetics. BMC Evol Biol.

[B56] Huelsenbeck JP, Ronquist F (2001). MRBAYES: Bayesian inference of phylogenetic trees. Bioinformatics.

[B57] Shimodaira H (2002). An approximately unbiased test of phylogenetic tree selection. Syst Biol.

[B58] Shimodaira H, Hasegawa M (2001). CONSEL: for assessing the confidence of phylogenetic tree selection. Bioinformatics.

