# Supplementary material for: Phylogeny and mitochondrial gene order variation in Lophotrochozoa in the light of new mitogenomic data from Nemertea
Source: BMC Genomics. 2009 Aug 6;10:364. doi: 10.1186/1471-2164-10-364 (PMC2728741; doi:10.1186/1471-2164-10-364)
Supplement: Additional file 1 — Accession numbers. [file 1471-2164-10-364-S1.doc]

Supplementary table 1. Species, systematic position and accession number of mitochondrial genome sequences used in the phylogenetic analysis and for gene order comparisons

| **Species** | **Taxonomic position** | **Accession no.** |
| --- | --- | --- |
| *Lineus viridis* | Nemertea | FJ839919 |
| *Cephalothrix rufifrons** | Nemertea | EF140788 |
| *Urechis caupo* | Echiura | NC_006379 |
| *Myzostoma seymourcollegiorum** | Myzostomida | EF506562 |
| *Lumbricus terrestris* | Annelida – Clitellata | NC_001677 |
| *Perionyx excavatus* | Annelida – Clitellata | NC_009631 |
| *Platynereis dumerilii* | Annelida – “Polychaeta“ | NC_000931 |
| *Orbinia latreillii* | Annelida – “Polychaeta“ | NC_007933 |
| *Eclysippe vanelli** | Annelida – “Polychaeta“ | EU239687 |
| *Clymenella torquata* | Annelida – “Polychaeta” | NC_006321 |
| *Pista cristata* | Annelida – “Polychaeta” | NC_011011 |
| *Terebellides stroemi* | Annelida – “Polychaeta” | NC_011014 |
| *Scoloplos armiger** | Annelida – “Polychaeta” | DQ517436 |
| *Phoronis psammophila** | Phoronida | AY368231 |
| *Terebratulina retusa* | Brachiopoda | NC_000941 |
| *Laqueus rubellus* | Brachiopoda | NC_002322 |
| *Terebratalia transversa* | Brachiopoda | NC_003086 |
| *Katharina tunicata* | Mollusca – Polyplacophora | NC_001636 |
| *Lottia digitalis* | Mollusca – Gastropoda | NC_007782 |
| *Haliotis rubra* | Mollusca – Gastropoda | NC_005940 |
| *Conus textile* | Mollusca – Gastropoda | NC_008797 |
| *Ilyanassa obsoloeta* | Mollusca – Gastropoda | NC_007781 |
| *Thais clavigera* | Mollusca – Gastropoda | NC_010090 |
| *Lophiotoma cerithiformis* | Mollusca – Gastropoda | NC_008098 |
| *Albinaria caerulea* | Mollusca – Gastropoda | NC_001761 |
| *Aplysia californica* | Mollusca – Gastropoda | NC_005827 |
| *Biomphalaria glabrata* | Mollusca – Gastropoda | NC_005439 |
| *Nautilus macromphalus* | Mollusca - Cephalopoda | NC_007980 |
| *Octopus ocellatus* | Mollusca - Cephalopoda | NC_007896 |
| *Venerupis phllippinarum* | Mollusca - Bivalvia | NC_003354 |
| *Argopecten irradians* | Mollusca - Bivalvia | NC_009687 |
| *Acanthocardia tuberculata* | Mollusca - Bivalvia | NC_008452 |
| *Crassostrea gigas* | Mollusca - Bivalvia | NC_001276 |
| *Loxocorone allax* | Entoprocta / Kamptozoa | NC_010431 |
| *Loxosomella aloxiata* | Entoprocta / Kamptozoa | NC_010432 |
| *Flustrellidra hispida* | Bryozoa / Ectoprocta | NC_008192 |
| *Bugula neritina* | Bryozoa / Ectoprocta | NC_010197 |
| *Paraspadella gotoi* | Chaetognatha | NC_006083 |
| *Spadella cephaloptera* | Chaetognatha | NC_006386 |
| *Brachionus plicatilis* | Syndermata – Rotifera | NC_010484 |
| *Leptorhynchoides thecatus* | Syndermata – Acanthocephala | NC_006892 |
| *Anisakis simplex* | Nematoda | NC_007934 |
| *Agamermis sp.* | Nematoda | NC_008231 |
| *Onchocercus volvulus* | Nematoda | NC_001861 |
| *Caenorhabditis elegans* | Nematoda | NC_001328 |
| *Trichinella spiralis* | Nematoda | NC_002681 |
| *Xiphinema americanum* | Nematoda | NC_005928 |
| *Microstomum lineare** | Platyhelminthes – “Turbellaria” | AY228756 |
| *Fasciola hepatica* | Platyhelminthes – “Trematoda” | NC_002546 |
| *Paragoniums westermanni* | Platyhelminthes – “Trematoda” | NC_002354 |
| *Gyrodactylus salaris* | Platyhelminthes – “Trematoda” | NC_008815 |
| *Microcotyle sebastis* | Platyhelminthes – “Trematoda” | NC_009055 |
| *Schistosoma haematobium* | Platyhelminthes – “Trematoda” | NC_008074 |
| *Trichobilharzia regenti* | Platyhelminthes – “Trematoda” | NC_009680 |
| *Diphyllobothrium latum* | Platyhelminthes – Cestoda | NC_008945 |
| *Hymenolepis diminuta* | Platyhelminthes – Cestoda | NC_002767 |
| *Taenia asiatica* | Platyhelminthes – Cestoda | NC_004826 |
| *Echinococcus granulosus* | Platyhelminthes – Cestoda | NC_008075 |
| *Priapulus caudatus* | Priapulida | NC_008557 |
| *Epiperipatus biolleyi* | Onychophora | NC_009082 |
| *Limulus polyphemus* | Chelicerata – Xiphosura | NC_003057 |
| *Centruroides limpidus* | Chelicerata – Scorpiones | NC_006896 |
| *Nothopuga* sp-1 LP-2008 | Chelicerata – Solifugae | NC_009984 |
| *Heptathela hangzhouensis* | Chelicerata – Araneae | NC_005924 |
| *Pseudocellus pearsei* | Chelicerata – Ricinulei | NC_009985 |
| *Ixodes hexagonus* | Chelicerata – Acari | NC_002010 |
| *Varroa destructor* | Chelicerata – Acari | NC_004454 |
| *Achelia bituberculata* | Pycnogonida | NC_009724 |
| *Narceus annularus* | Myriapoda – Diplopoda | NC_003343 |
| *Lithobius forficatus* | Myriapoda – Chilopoda | NC­_002629 |
| *Petrobius brevistylis* | Hexapoda – Archaeognatha | NC_007689 |
| *Tricholepidion gertschi* | Hexapoda – Zygentoma | NC_005437 |
| *Periplaneta fuliginosa* | Hexapoda – Blattodea | NC_006076 |
| *Locusta migratoria* | Hexapoda – Orthoptera | NC_001712 |
| *Apis mellifera* | Hexapoda – Hymenoptera | NC_001566 |
| *Drosophila yakuba* | Hexapoda – Diptera | NC_001322 |
| *Tribolium castaneum* | Hexapoda – Coleoptera | NC_003081 |
| *Speleonectes tulumensis* | Crustacea – Remipedia | NC_005938 |
| *Argulus americanus* | Crustacea – Branchiura | NC_005935 |
| *Armillifer armillatus* | Crustacea – Pentastomida | NC_005934 |
| *Artemia franciscana* | Crustacea – Anostraca | NC_001620 |
| *Triops* *cancriformis* | Crustacea – Phyllopoda | NC_004465 |
| *Penaeus* *monodon* | Crustacea – Decapoda | NC_002184 |
| *Pagurus longicarpus* | Crustacea – Decapoda | NC_003058 |
| *Squilla mantis* | Crustacea – Stomatopoda | NC_006081 |
| *Ligia oceanica* | Crustacea – Peracarida | NC_008412 |
| *Balanoglossus carnosus* | Enteropneusta | NC_001887 |
| *Saccoglossus kowalevskii* | Enteropneusta | NC_007438 |
| *Florometra serratissima* | Echinodermata – Crinoidea | NC_001878 |
| *Paracentrotus lividus* | Echinodermata – Echinoidea | NC_001572 |
| *Arbacia lixula* | Echinodermata – Echinoidea | NC_001770 |
| *Ophiura lutkeni* | Echinodermata – Ophiuroidea | NC_005930 |
| *Asterias amurensis* | Echinodermata – Asteroidea | NC_006665 |
| *Cucumaria miniata* | Echinodermata – Holothuroidea | NC_005929 |
| *Branchiostoma floridae* | Chordata – Acrania | NC_000834 |
| *Lampetra fluviatilis* | Chordata – Vertebrata | NC_001131 |
| *Petromyzon marinus* | Chordata – Vertebrata | NC_001626 |
| *Eptatretus burgeri* | Chordata – Vertebrata | NC_002807 |
| *Myxine glutinosa* | Chordata – Vertebrata | NC_002639 |
| *Xenoturbella bocki* | Xenoturbellida | NC_008556 |
| *Acropora tenuis* | Cnidaria – Anthozoa | NC_003522 |
| *Briareum asbestinum* | Cnidaria – Anthozoa | NC_008073 |
| *Pseudopterogorgia bipinnata* | Cnidaria – Anthozoa | NC_008157 |
| *Aurelia aurita* | Cnidaria – Scyphozoa | NC_008446 |
| *Geodia neptuni* | Porifera | NC_006990 |
| *Amphimedon queenslandica* | Porifera | NC_008944 |
| *Oscarella carmela* | Porifera | NC_009090 |

* : incomplete genome sequence
